# Statistical analysis and a case study of tropical cyclones that trigger the onset of the South China Sea summer monsoon

**DOI:** 10.1038/s41598-017-13128-2

**Published:** 2017-10-06

**Authors:** Jingliang Huangfu, Ronghui Huang, Wen Chen

**Affiliations:** Center for Monsoon System Research, Institute of Atmospheric Physics, Chinese Academy of Sciences, Beijing, China

## Abstract

This paper addresses whether a tropical cyclone can trigger the onset of the South China Sea (SCS) summer monsoon (SM). We conducted a statistical analysis of tropical cyclones (TCs) generated over the western North Pacific (WNP) between late-April and May. The results showed that there were cases in which TCs were generated before the onset of the SCSSM, accounting for 43.2% of the TCs generated during this season. This study examined a representative case, Super Typhoon Chanchu (0601), which was determined to be influential in the onset of the SCSSM. With a northwestward track, Chanchu brought strong convection and westerly winds to the SCS on 12 May, which triggered the intrusion of the southwesterly winds from the Bay of Bengal and the eastward retreat of the western Pacific subtropical high. Super Typhoon Chanchu provides an example in which a TC triggered the onset of the SCSSM. The negative correlation between the onset date of the SCSSM and the number of TCs generated over the WNP used to be interpreted as the influence of the monsoon trough on TC genesis. This work provides a supplementary illustration that this relationship also includes the impact of TCs on the onset of the SCSSM.

## Introduction

Broadly defined, the East Asian summer monsoon onset starts in the South China Sea (SCS)^1^. The entrance of the prevailing southwesterly winds into the SCS region marks the establishment of the summer monsoon (SM), which brings the rainy season to South China and carries water vapor from the Bay of Bengal^2^. Therefore, the onset date of the South China Sea summer monsoon (SCSSM) has significant influences on the East Asian summer precipitation and can be used as a very important indicator for short-term climate predictions in the countries of East Asia^3–5^.

Previous studies have shown that there are multiple time scale variations in the onset date of the SCSSM^6–12^. The variations are related to the thermal states of the Pacific, including the El Niño–Southern Oscillation (ENSO), the Pacific Decadal Oscillation (PDO) and the changes over the western Pacific^13–15^. Several other studies have stressed that the influence of the Indian Ocean is of equal importance^16,17^. The roles played by these two oceans may change under different climate regimes^18^. There are also studies that have investigated the land-sea thermal differences^19,20^. Additionally, some studies have argued that the onset of the SCSSM is more local than expected, with the SCS thermal state mainly implicated^21^.

The interannual variation of the SCSSM receives considerable attention from the meteorological departments and governments, due to its implications on the aforementioned precipitation predictions. Recently, Huangfu *et al*.^22,23^ investigated the relationship between the onset date of the SCSSM and tropical cyclone (TC) genesis over the western North Pacific (WNP) in May. The interannual SCSSM onset is significantly correlated with the number of WNP TCs generated^23^. The authors interpreted the negative correlation as an indication that the onset of the SCSSM may influence the WNP TC genesis by affecting the eastward extension of the monsoon trough. Chen *et al*.^24^ further described this process and developed a short-term forecast advisory. They noted that the onset of the SCSSM is almost coincident with the start of the WNP typhoon season. Moreover, it is interesting to address whether this correlation also indicates that TCs can trigger the onset of the SCSSM. As we know, the onset of the SCSSM is characterized by a sudden outbreak of onset, with limited forecast accuracy. Webster and Yang^13^ proposed that the circulation over the SCS region during this period is very sensitive to forcings that can disturb the wind strength in the tropics. Hence, although the energy brought by a single TC cannot match that of a summer monsoon, it may act as a bomb to a breaking dam. Therefore, it is meaningful to count the number of WNP TCs that have been generated before the onset of the SCSSM and examine their paths, which may be critical to the establishment of the SCSSM.

## Results

### The statistical analysis of the WNP TCs generated before the onset of the SCSSM

**Figure 1:**
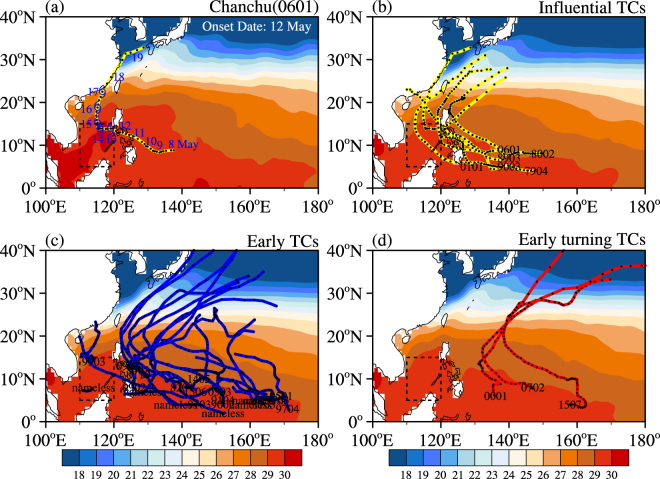
Observed moving paths of (**a**) Super Typhoon Chanchu (yellow line), (**b**) influential TCs, (**c**) early TCs and (**d**) early turning TCs, which are marked with black dots every 0.25 days. The typhoon symbols in figure (**a**) represent the period when the intensity of the tropical cyclone exceeded the typhoon level. The Hadley SSTs (units: °C) in (**a**) May 2006, (**b**–**d**) April–May were averaged over the respective years and are shaded. The dashed black boxes denote the SCS region (5–15°N, 110–120°E). The maps in the figure are generated using the NCL software (Version: 6.4.0 & URL: http://www.ncl.ucar.edu/).

To analyze the influence of TCs on the onset of the SCSSM, we counted the interannual TC genesis numbers over the WNP region (0°–30°N, 110°E–180°) from mid-April to May for the period 1979–2015 (Table 1). There are 30 years during this period in which a total of 74 WNP TCs were generated, and the 7 years without TCs are not listed in Table 1. The onset date of the SCSSM was determined according to the definition of Wang *et al*. (2004) and the ERA-Interim Reanalysis dataset, and significant interannual variations were observed. According to our statistics, there are 23 years when TCs were generated before the onset of the SCSSM, 32 TCs in total, accounting for 43.2% of the TCs formed between Mid-April and May. Further, as shown in Fig. 1b–d, the TCs generated prior to the onset of the SCSSM were classified into three groups mostly considering their movements: influential, early and early turning path TCs. The influential TCs were identified as passing by or near the SCS region just before the onset of the SCSSM. The early TCs were generated too early to trigger the onset of the monsoon. The early turning path TCs turned northeastward when they reached the eastern Philippines and may not have been responsible for the onset of the SCSSM. Among the 32 TCs, 6 were identified as influential and are identified in bold font in Table 1. As mentioned above, the circulation over the SCS region is very sensitive to forcings over the tropics. Hence, although there is a large discrepancy in the energy brought by the influential TCs, their effects seem similar. For example, tropical storm Cimaron (0101) triggered the onset of the SCSSM in 2001 despite its weak maximum sustained wind (MSW) speed and short duration. More details about Cimaron are available in Text SM1. The moving path and the timing of the TCs generated before the onset of the SCSSM seem to carry more importance. The investigation of the influential TCs may help answer the question as to whether a tropical cyclone can trigger the onset of a monsoon.

**Table 1:** The information of the WNP TCs from mid-April to May over the period from 1979–2015.

Year	TC Num1	SCSSM onset date	TC Num2	Genesis Information	Classification
1979	3	27 (pentad)	1	50604148	*.Dot7904
1980	4	27	1	50508150	*.Dom8002
1981	1	31	1	42804164	1.Holly8103
1982	1	31	1	51608140	1.Pat8204
1985	2	30	2	42012127; 51607129	1.nameless; 1.Gay8503
1986	3	27	1	42202152	1.Ken8602
1988	1	29	0	none	none
1989	2	28	1	51407138	*.Brenda8903
1990	4	28	2	42704152; 50905138	1.Lewis9002; *.Marine9003
1991	2	32	2	42104145; 50405152	1.Vanessa9103; 1.Walt9104
1993	2	32	2	41805161; 51404160	1.nameless; 1.nameless
1994	2	25	0	none	none
1995	4	27	2	42306169; 42805164	1.Chuck9501; 1.nameless
1996	3	26	1	42508114	1.nameless
1997	4	28	2	41805168; 50403171	1.Jimmy9703; 1.Kelly
1999	3	30	2	42108126; 42614114	1.Kate9902; 1.Leo9903
2000	4	26	1	50406136	2.Damrey0001
2001	2	26	2	41604138; 50505129	1.nameless; *.Cimaron0101
2002	2	27	0	none	none
2003	4	28	0	none	none
2004	3	27	0	none	none
2005	2	26	1	41707151	1.Sonca0503
2006	1	27	1	50809138	*.Chanchu0601
2007	1	29	1	51508147	2.Yutu0702
2008	4	25	0	none	none
2009	2	29	1	50113124	1.Kujira0901
2011	3	29	1	50611129	1.Aere1101
2012	2	25	0	none	none
2014	1	32	1	42710145	1.Tapah1405
2015	2	28	2	50207144; 50704158	1.Noul1506; 2.Dolphin1507

Classification: *influential TCs, 1 early TCs, 2 early turning path TCs.

The numbers in column 2 are the numbers of TCs which were generated between Mid-April and May. The numbers in column 4 are the numbers of TCs which were generated before the onset of the SCSSM.

Genesis Information is shown with an eight-bit number (MDDTTNNN): M is the corresponding month, DD is the day of month, TT is the latitude and NNN is the longitude of the first recorded observation of the storm.

### A case study of Chanchu (0601)

After considering the strength and moving path of multiple TCs, this study selected the Super Typhoon Chanchu (0601) as a representative case. As shown in Fig. 1a, the tropical SST in May 2006 was over 29 °C, and the temperature over the SCS region was even higher. Under such an oceanic thermal state, Chanchu was generated around 10°N, 140°E and was first recorded on 7 May^25^. With a northwestward track, Chanchu reached the northern SCS region on 12 May and developed into a typhoon on 13 May. Later, Chanchu made a sharp northeastern turn on 16 May, which may be related to the onset of the SCSSM.

**Figure 2:**
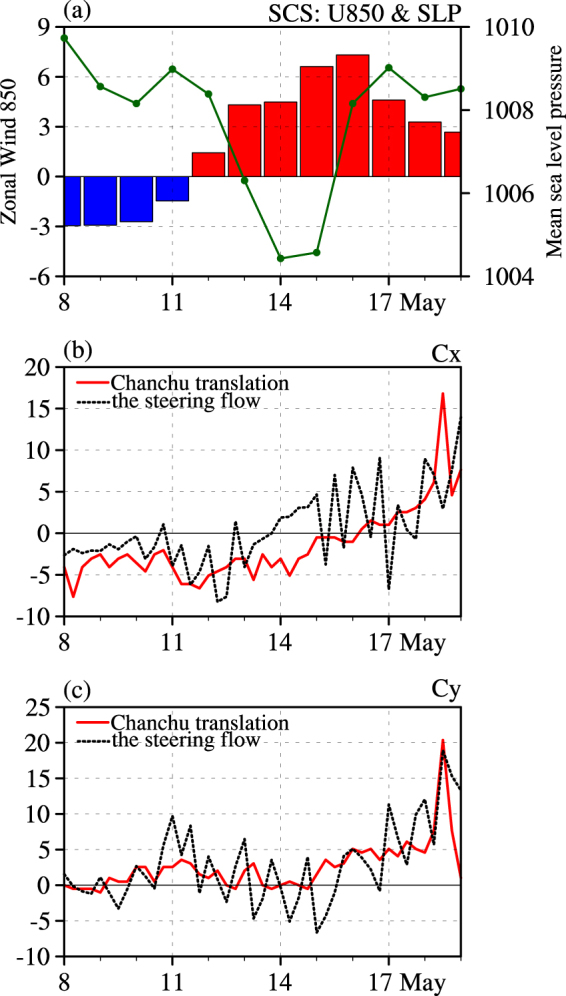
Time series of (**a**) the regional average 850 hPa horizontal zonal winds (bars, units: m s^−1^) and the mean sea level pressure (solid green line) over the SCS (5–15°N, 110°–120°E) during 7–19 May 2006, (**b**) the zonal components Cx (unit: m s^−1^) and (**c**) the meridional components Cy (units: m s^−1^) of the translation speed of Typhoon Chanchu (solid red line) and the steering flow (dashed black line).

Based on the ERA-interim daily data as shown in Fig. 2a, the influence of Super Typhoon Chanchu on the onset of the SCSSM can be partially inferred. The regional average winds over the SCS became positive (westerly) after 12 May when Chanchu reached the eastern SCS. The curve of the sea level pressure (SLP) clearly reflects the movement of Chanchu. A rapid decline in the SLP began on 12 May, and the SLP of the SCS reached its minimum on 14 May when Chanchu moved into the northern SCS (Fig. 1a). The westerly winds related to Super Typhoon Chanchu continuously increased from 12 to 16 May. Although the westerly winds became weaker after the typhoon, they remained over the SCS. In addition, we provided objective evidence on the translation of Chanchu. Previous studies have shown that a TC is largely influenced by the steering flow, including large-scale environmental flow and the ventilation flow^25–29^. Following Wu *et al*.^30^, the steering flow is calculated as the mass-weighted mean wind averaged within a radius of 440 km (4° latitudes) between 850 and 300 hPa. As shown in Fig. 2, the calculated steering is reasonably consistent with the translation speeds of the typhoon. The steering flow carried Chanchu westward during 8–12 May (Fig. 2b). Typhoon Chanchu followed the easterly trade winds to the south of the western Pacific subtropical high (WPSH) before it reached the SCS. This indicated that the WPSH could be treated as the most important dynamic system that affects the typhoon motion, which is consistent with the conclusion of Wu *et al*.^31^. The steering flow became eastward on 12 May, when the onset of SCSSM was triggered and the southwesterly monsoon became a more important dynamic system that affected the movement of Chanchu. In the meridional direction (Fig. 2c), the steering flow first enhanced the northward movement of Chanchu during 10–12 May and then 15–19 May. The “L-type” typhoon track of Chanchu was influenced by these two dynamic systems.

**Figure 3:**
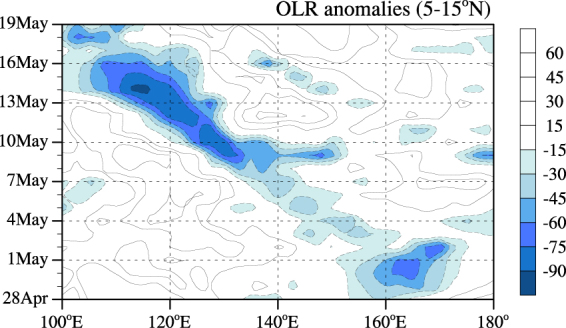
The evolution of the meridional mean (5–15°N) OLR anomalies (units: Wm^−2^) from 28 April–19 May 2006.

As shown in Fig. 3, the evolution of the meridional mean (5°–15°N) OLR anomalies is analyzed to obtain more information about the onset process of the SCSSM in 2006. The most active convective activity began to the west of 140°E and was mostly related to the Chanchu genesis on 7 May. The deep convection induced by Chanchu propagated northwestward after the TC was generated, which is consistent with the typhoon path as shown in Fig. 1. This strong convection entered the SCS region on 10 May and reached its peak on 14 May. The evolution of the OLR anomalies showed that Chanchu was the main weather process during the period before the onset of the SCSSM, and Chanchu should be considered the most influential factor in the onset process.

**Figure 4:**
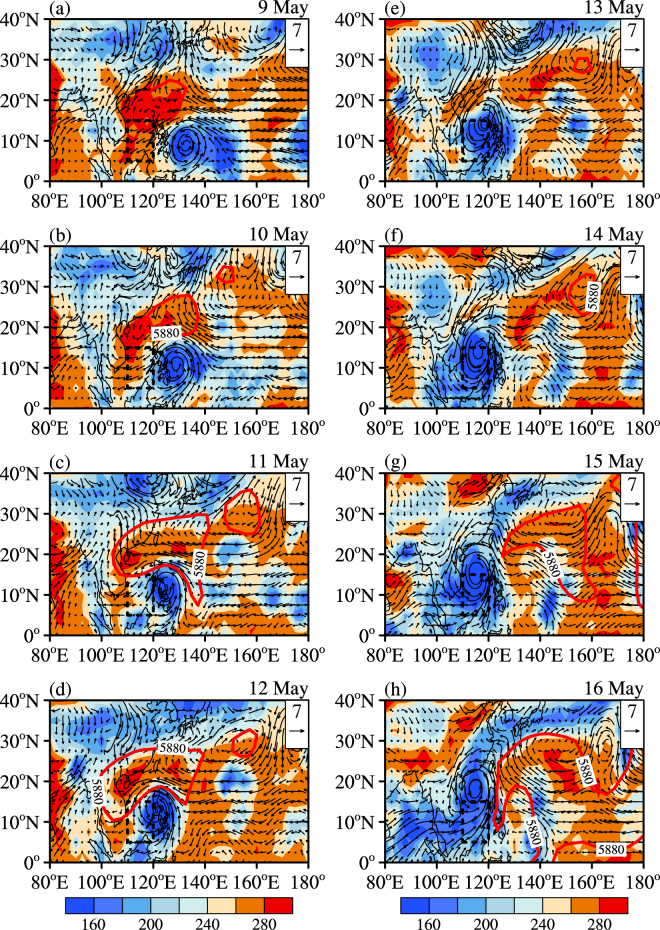
The 850 hPa horizontal wind field (vector, units: m s^−1^), the OLR (shading, units: Wm^−2^) and the 500 hPa 5880 gpm line (solid red line) from 9 to 16 May 2006. The dashed black box denotes the SCS region (5–15°N, 110–120°E). The maps in the figure are generated using the NCL software (Version: 6.4.0 & URL: http://www.ncl.ucar.edu/).

We further analyzed the evolutionary characteristics of the low-level (850 hPa) wind field and the WPSH (represented by the 500 hPa 5880 gpm line) from 9 to 16 May (Fig. 4). It is shown that the WPSH controlled the SCS region before 10 May. The main part of the WPSH moved out of the SCS on 11 May, but its southern portion still controlled this region, as the easterly trade winds prevailed in this area. Chanchu intruded into the SCS on 12 May, and its western part brought westerly winds to the SCS. Since then, the WPSH withdrew from the SCS region and moved eastward. The westerly winds to the south of Super Typhoon Chanchu triggered the intrusion of the southwesterly winds from the Bay of Bengal, which joined the southern part of Typhoon Chanchu on 14 May. This process maintained the westerly winds over the central SCS after the typhoon and helped establish the SCSSM. Typhoon Chanchu provides a typical example in which a TC triggered the onset of the SCSSM.

## Summary and Discussion

This study conducted a statistical analysis of the WNP TCs generated before the onset of the SCSSM. During the period from 1979–2015, there were 30 years in which WNP TCs were generated between Mid-April and May, with 74 TCs in total. A total of 43.2% of these TCs were generated before the onset of the SCSSM. To analyze the influence of TCs on the onset of the SCSSM, the TCs that occurred prior to the onset of the SCSSM were classified into three groups by mostly considering their movements. The influential TCs were identified as those passing by or near the SCS region just before the onset of the SCSSM, which may help answer the question as to whether a tropical cyclone can trigger the onset of a monsoon.

This study selected a representative case, Super Typhoon Chanchu (0601), which passed by the SCS region just before the establishment of the SCSSM. The results showed the evolutionary characteristics of the onset of the SCSSM process. The deep convection that was induced by Chanchu propagated northwestward after the TC was generated (7 May), which was consistent with the path of Chanchu. Chanchu brought strong cyclonic circulation to the SCS region and the most active convective activity intruded the SCS on 12 May, when Chanchu entered this area. Afterward, the WPSH began to withdraw eastward. The westerly winds to the south of Super Typhoon Chanchu triggered the intrusion of the southwesterly winds from the Bay of Bengal on 14 May, which helped establish the SCSSM. Super Typhoon Chanchu provides a typical example in which a TC triggered the onset of the SCSSM.

This work conducted a supplementary analysis of the correlation between the onset of the SCSSM and WNP TCs^32^. The unilateral understanding of the influence of the monsoon trough on TC genesis is not sufficient to interpret their relationship. This result filled this knowledge gap by providing a statistical analysis and case study that showed the impact of TCs on the onset of the SCSSM. However, more integral work should be carried out in the near future, including comparisons between the years in which there were influential TCs and the years without preceding TCs, numerical experiments investigating to what extent a TC can accelerate the onset of the SCSSM and other meaningful analyses. In addition, it is important to investigate the oceanic conditions during influential TCs, as this may be helpful in interpreting their paths of movement.

## Methods

### Data

The WNP TC genesis locations and the paths of the TCs are analyzed using the TC best track data (v03r09) from the International Best Track Archive for Climate Stewardship (IBTrACS)^33^ for the time period from 1979–2015. Moreover, to analyze the Super Typhoon Chanchu, the daily horizontal winds at 850 hPa, the geopotential height at 500 hPa and the mean sea level pressure are extracted from the European Centre for Medium-Range Weather Forecasts Interim (ERA-Interim) reanalysis dataset^34^. The MSW data recorded in IBTrACS for Chanchu are from four agencies: the Joint Typhoon Warning Center, the China Meteorological Administration - Shanghai Typhoon Institute, the RSMC Tokyo and the Hong Kong Observatory. The average value of the MSW from the four observations reached 67.25 knots on 2006051312, which is considered typhoon intensity. In addition, this study employs the daily outgoing longwave radiation (OLR) data from the National Oceanic and Atmospheric Administration (NOAA) archives during Super Typhoon Chanchu^35^. The monthly mean sea surface temperature data from the Met Office Hadley Centre are used for the oceanic background conditions^36^. The resolutions of the ERA-interim data and the OLR data are 2.5° × 2.5°, and that for the SST data is 1° × 1°.

## Electronic supplementary material

Supplementary Information
