# Supplementary material for: Statistical analysis and a case study of tropical cyclones that trigger the onset of the South China Sea summer monsoon
Source: Sci Rep. 2017 Oct 6;7:12732. doi: 10.1038/s41598-017-13128-2 (PMC5630633; doi:10.1038/s41598-017-13128-2)
Supplement: Supplementary file 1 — Supplementary Information [file 41598_2017_13128_MOESM1_ESM.doc]

**Supplementary Information (SM)**

**Statistical analysis and a case study of tropical cyclones that trigger the onset of the South China Sea summer monsoon**

Jingliang Huangfu^1^*, Ronghui Huang^1^, Wen Chen^1^

^1^Center for Monsoon System Research, Institute of Atmospheric Physics, Chinese Academy of Sciences, Beijing, China.

^*^**Corresponding Author:**

Jingliang Huangfu

Institute of Atmospheric Physics

Chinese Academy of Sciences

Beijing 100190, China

Ph: +86 010 62522491

e-mail: hfjl@mail.iap.ac.cn

**Supplementary Figures.**

**Figure SM1**. Observed path (yellow line) of Tropical Storm Cimaron (0101) marked with black dots. The Hadley SSTs (units: °C) in May are shaded. The dashed black boxes denote the SCS region (5–15°N, 110–120°E). The maps in the figure are generated using the NCL software (Version: 6.4.0 & URL: http://www.ncl.ucar.edu/).

**Figure SM2.** The evolution of the meridional mean (5–15°N) OLR anomalies (units: Wm^-2^) during Tropical Storm Cimaron (0101).

**Figure SM3**. Time series of the regional average 850 hPa horizontal zonal winds (bars, units: m s^-1^) and the mean sea level pressure (solid green line) over the SCS (5–15°N, 110°–120°E) during Tropical Storm Cimaron (0101).

**Text SM1**

**Maximum sustained winds (MSW) Information:**

**The Cimaron (0101) data in IBTrACS are from four agencies: the Joint Typhoon Warning Center (JTWC), the Shanghai Typhoon Institute (STI), the RSMC Tokyo and the Hong Kong Observatory (HKO).**

|  | TIME | JTWC | STI | Tokyo | HKO | Average |
| --- | --- | --- | --- | --- | --- | --- |
| CIMARON | 2001050500 | 25 | -9990 | -9990 | -9990 |  |
| CIMARON | 2001050506 | 20 | -9990 | -9990 | -9990 |  |
| CIMARON | 2001050512 | 20 | -9990 | -9990 | -9990 |  |
| CIMARON | 2001050518 | 20 | -9990 | -9990 | -9990 |  |
| CIMARON | 2001050600 | 20 | -9990 | -9990 | -9990 |  |
| CIMARON | 2001050606 | 20 | -9990 | -9990 | -9990 |  |
| CIMARON | 2001050612 | 20 | -9990 | -9990 | -9990 |  |
| CIMARON | 2001050618 | 20 | -9990 | -9990 | -9990 |  |
| CIMARON | 2001050700 | 25 | -9990 | -9990 | -9990 |  |
| CIMARON | 2001050706 | 25 | -9990 | -9990 | -9990 |  |
| CIMARON | 2001050712 | 25 | -9990 | -9990 | -9990 |  |
| CIMARON | 2001050718 | 25 | -9990 | -9990 | -9990 |  |
| CIMARON | 2001050800 | 25 | -9990 | -9990 | -9990 |  |
| CIMARON | 2001050806 | 25 | -9990 | -9990 | -9990 |  |
| CIMARON | 2001050812 | 25 | -9990 | -9990 | -9990 |  |
| CIMARON | 2001050818 | 25 | -9990 | -9990 | -9990 |  |
| CIMARON | 2001050900 | 25 | -9990 | -9990 | -9990 |  |
| CIMARON | 2001050906 | 30 | -9990 | -9990 | -9990 |  |
| CIMARON | 2001050912 | 30 | 23 | -9990 | -9990 |  |
| CIMARON | 2001050918 | 30 | 23 | -9990 | -9990 |  |
| CIMARON | 2001051000 | 35 | 23 | -9990 | -9990 |  |
| CIMARON | 2001051006 | 35 | 29 | -9990 | 25 |  |
| CIMARON | 2001051012 | 40 | 29 | -9990 | 25 |  |
| CIMARON | 2001051018 | 40 | 29 | -9990 | 25 |  |
| CIMARON | 2001051100 | 40 | 29 | 35 | 30 | 31.6 |
| CIMARON | 2001051106 | 40 | 35 | 35 | 35 | 34 |
| CIMARON | 2001051112 | 45 | 35 | 35 | 35 | 35.2 |
| CIMARON | 2001051118 | 45 | 39 | 40 | 40 | 38.2 |
| CIMARON | 2001051200 | 45 | 45 | 40 | 40 | 39.6 |
| CIMARON | 2001051206 | 45 | 45 | 40 | 40 | 39.8 |
| CIMARON | 2001051212 | 45 | 45 | 40 | 40 | 40 |
| CIMARON | 2001051218 | -9990 | -9990 | 45 | -9990 |  |
| CIMARON | 2001051300 | 50 | 45 | 45 | 45 |  |
| CIMARON | 2001051306 | -9990 | -9990 | 45 | -9990 |  |
| CIMARON | 2001051312 | 50 | 45 | 50 | 45 |  |
| CIMARON | 2001051318 | -9990 | -9990 | 50 | -9990 |  |
| CIMARON | 2001051400 | 50 | 49 | 45 | 50 |  |
| CIMARON | 2001051406 | -9990 | -9990 | 45 | -9990 |  |
| CIMARON | 2001051412 | 55 | 49 | 45 | 50 |  |
| CIMARON | 2001051418 | -9990 | -9990 | 45 | -9990 |  |
| CIMARON | 2001051500 | 60 | 49 | 45 | 50 |  |
| CIMARON | 2001051506 | -9990 | -9990 | 45 | -9990 |  |
| CIMARON | 2001051512 | 55 | 49 | 45 | 50 |  |
| CIMARON | 2001051518 | -9990 | -9990 | 45 | -9990 |  |
| CIMARON | 2001051600 | 45 | 39 | 40 | 40 |  |
| CIMARON | 2001051606 | -9990 | -9990 | 35 | -9990 |  |
| CIMARON | 2001051612 | 50 | 35 |  | 35 |  |
| CIMARON | 2001051618 |  | 29 |  |  |  |
| CIMARON | 2001051700 |  | 29 |  |  |  |
| CIMARON | 2001051706 |  | 29 |  |  |  |
| CIMARON | 2001051712 |  | 23 |  |  |  |
